# Determinants of Primary School Non-Enrollment and Absenteeism: Results from a Retrospective, Convergent Mixed Methods, Cohort Study in Rural Western Kenya

**DOI:** 10.1371/journal.pone.0138362

**Published:** 2015-09-15

**Authors:** Nia King, Cate Dewey, David Borish

**Affiliations:** 1 Department of Population Medicine, Ontario Veterinary College, Guelph, Ontario, Canada; 2 Centre for Public Health and Zoonoses, University of Guelph, Guelph, Ontario, Canada; TNO, NETHERLANDS

## Abstract

**Background:**

Education is a key element in the socioeconomic development required to improve quality of life in Kenya. Despite the introduction of free primary education, primary school enrollment and attendance levels remain low. Drawing on qualitative and quantitative data, this study explores the determinants of non-enrollment and absenteeism in rural western Kenya and potential mitigation strategies to address these issues.

**Methods:**

The study was conducted in Bwaliro village in rural western Kenya. A random sample of 64 students was obtained by blocking the village primary school’s student population according to grade level, gender, and orphan status. Qualitative and quantitative data were collected through interviews with parents, guardians, and key informants, and focus group discussions with students. Quantitative data were compared using chi-square tests, Student’s T-test, and Poisson regressions. Qualitative data were analyzed using thematic content analysis.

**Results:**

Malaria, menstruation, and lack of money were among the most notable determinants of primary school dropout and absenteeism, and these factors disproportionately impacted orphans and female students. Potential mitigation strategies suggested by the community included provision of malaria treatment or prevention, reduction in education costs, expansion of the established school-feeding program, and provision of sanitary pads.

**Conclusion:**

Despite free primary education, numerous factors continue to prevent children in rural western Kenya from attending primary school. The findings suggest that interventions should primarily target orphaned and female students. Prior to implementation, suggested mitigation strategies should be assessed for cost-effectiveness.

## Introduction

Education is a prerequisite to the socioeconomic development needed to alleviate poverty and improve quality of life. With literacy levels of 90% for men and 83% for women, Kenya is more literate than Sub-Saharan Africa as a whole, which has average levels of 76% for men and 63% for women]. However, with a third of the population having incomplete primary education, Kenya’s labour force lacks the education required to reach its Vision 2030 goals [1–2]. In September 2000, the Kenyan government signed the Millennium Development Goals (MDGs), aimed to combat the many dimensions of poverty, and subsequently developed the Kenya Vision 2030, which aims to make Kenya a middle-income country by 2030 [3–4]. School attendance is an important proxy for educational outcomes; by improving access to education, Kenya would make progress toward achieving both the Vision 2030 Plan and several MDGs including achieving universal primary education, improving food security, promoting gender equality, reducing child mortality, improving maternal health, and reducing the levels of HIV/AIDS and malaria [2–3,5–7].

The Kenyan government views education as the primary means of socioeconomic development and has therefore identified the challenges to achieving universal primary education [4]. These include: limited community participation, inadequate nutrition and health support services, high drop-out rates, imposition of school levies and other fees, cultural prejudice and negative attitudes towards Orphan and Vulnerable Children (OVCs), and increased numbers of OVCs [4,8–9]. In 2003, the Kenyan government introduced free primary education to cover tuition, however families still shoulder the costs of uniforms, activities, exams, and tuition for the Early Childhood Development (ECD) classes for three to five year old children, which are substantial expenses for poor families with multiple school-aged children [10–14]. Thus, despite the government’s 2012 goal of reducing non-enrollment to 5%, it still stands at 9.1%, in part, due to these costs [15].

Previous studies in Kenya and other Sub-Saharan countries have found that girls, rural children, and impoverished children are at increased risk of being unenrolled [12,16]. As guardians pressure girls to marry because of dowry payments and having fewer dependents, early marriage and teenage pregnancy play significant roles in the high female dropout level [6,10,17]. Gender stereotyping, such as the belief that women do not require an education as they belong in the house supporting the family, can also play a role in the higher dropout level for females [12]. The added responsibilities that orphans must take on, including financial, food, and childcare responsibilities, increase orphans’ dropout risk [1,18–21].

In addition to low enrollment and high dropout levels, absenteeism must be addressed given 11.4% of enrolled Kenyan children were absent on any given day in 2012 [15]. Cost of schooling, parental influence, marriage, pregnancy, menstruation, and household chores are recognized contributors to absenteeism [12,16,22–23]. Lastly, among the preventable medical causes of absenteeism, malaria infection accounts for 13% to 50% of school days missed in Kenya [24].

Various interventions aiming to improve primary school enrollment and attendance have been tested. Comprehensive school support, including food supplementation, school fees, uniforms, and a school-based helper, for orphans in Kenya and Zimbabwe significantly reduced dropout and absence rates [6,10,18]. The provision of free sanitary pads to schoolgirls in Ghana and Kenya has improved girls’ attendance levels, concentration, and confidence [25–27]. Halliday et al. (2014) found that school-based intermittent malaria screening and treatment was not effective in improving the health or education of school children, while Aikins (1995) found that impregnating bed nets with insecticides reduced absenteeism due to ill-health [28–29]. In Chile, Dinkelman and Martinez (2014) found that providing students with information about the opportunities available to finance higher education effectively improved primary school attendance [30]. Given the widely recognized positive impacts of school-based feeding programs on school attendance and learning achievements, such programs are beginning to be implemented in many developing countries [31].

While some programs have successfully reduced absenteeism, to the authors’ knowledge, no studies have examined the perceived benefits of education within rural Kenyan communities. There is also little knowledge regarding the determinants of dropouts and absenteeism in rural western Kenya, without which it is difficult to develop effective community-based strategies to address these issues. We therefore examined the community’s perceptions of the benefits of education and the main factors contributing to high non-enrollment and absenteeism levels in Bwaliro village of rural western Kenya. The community’s input regarding possible mitigation strategies was also sought. The findings will be useful to policy makers and stakeholders to develop optimal strategies to improve enrollment and attendance levels in rural western Kenya.

## Methods

### Study Area and Population

This study was conducted in Bwaliro village, located in Busia County of western Kenya between May and July 2014. Bwaliro village lies in one of Kenya’s poorest regions: 66.7% of Busia County’s population lives below the poverty line and 76% is food insecure [32–33]. Subsistence farming is the main economic activity; all families rely on their plot of farming land, or *shamba*, as a food and/or income source. Western Kenya has the second highest HIV/AIDS prevalence (6.6%) in all Kenyan regions, resulting in a large orphan population who is at increased risk of dropping out of school or being absent [1–2,34]. Malaria was the most common disease treated at the local dispensary in patients over the age of five years.

This study was centered on Bwaliro village’s public primary school: Bwaliro Primary School. School administrators have identified approximately 35% of the Bwaliro students as OVCs. In 2010, the Children of Bukati Organization partnered with the school to establish a feeding and agro-forestry program [35]. All students from ECD to grade 3, in addition to OVCs from grades 4 to 8, are fed a lunch of *githeri* (boiled maize and beans) on a triweekly basis. Additionally, students in grade 8 bring in their own food to cook a daily communal lunch. Yields from the agro-forestry program are used to subsidize the feeding program. Many children travel from neighbouring villages to be involved in these programs.

### Recruitment and Participants

We conducted a retrospective, convergent mixed methods, cohort study [36]. Students from grades 1 to 8 were blocked by grade, OVC status, and gender. Four students from each block were selected, including two replacements, using simple random sampling as follows. Teachers provided class lists divided by OVC status and gender. Students were numbered and four random numbers per block were selected. If multiple students who cited the same guardian were chosen, only one was included, and other randomly chosen students replaced the excluded ones. The researchers visited the selected students’ households. Upon arrival, the interpreter explained the project and asked if the parent or guardian (henceforth referred to as guardians) was willing to participate. The first two students in each block whose guardian was available and willing to be interviewed were included, yielding a total of 64 students (32 OVCs and 32 NOVCs). The sample size of 64 guardians was based on an estimate of 0.7 days absent for NOVCs and of 1.6 days for OVCs. We expected a similar difference between boys and girls, respectively. Based on a variance of 1.2 days, a power of 80%, and a significance level of 0.05, approximately 28 participants were required in each category. Female guardians were interviewed, as traditionally females are responsible for childcare. If there was no female adult living in the household, the adult male was interviewed. OVCs were categorized based on which parent died, and guardians were categorized based on their relationship with the child. All guardians were willing to participate. No compensation was offered.

Key informant interviews were conducted with the principal, four teachers, the village chief, the District Education Officer, a church official, and two local nurses. The 24 grade 6 to 8 students whose guardians had been interviewed were invited to participate in focus group discussions (FGDs). All of these students participated.

### Interview Approach

The data were collected using a household questionnaire through a local interpreter who spoke either Kimarachi (the local vernacular language) or Kiswahili, depending on the guardian’s preference. The questionnaire was written by the authors in English. Questions were translated into both Kimarachi and Kiswahili by one interpreter and were then back translated by another interpreter to ensure that the meaning of the question was maintained. The questionnaire was piloted in 20 randomly selected village households; appropriate changes were made thereafter. The opening questions addressed independent variables. Household-level variables were: self-reported household income and number of days the randomly chosen child was absent from school within the previous two weeks. Guardian level variables were: age, gender, and level of education. Next, the questionnaire asked open-ended questions regarding perceived level of community support for education, perceived benefits of education, determinants of dropout and absenteeism, potential mitigation strategies, and the impacts of pre-established programs on students’ education.

During the interview process, the interpreter would ask the question, listen to the answer, and then translate the response for the first author who transcribed the answers. Interviews were conducted within the guardian’s compound and typically lasted between 30 and 40 minutes.

Key informant interviews were conducted in English, since all respondents were fluent in English. Key informants were asked about the level of community support for education, perceived benefits of education, determinants of dropout and absenteeism, potential mitigation strategies, and observed impacts of pre-established school programs. These interviews were conducted in the informant’s office, classroom, or home, depending on the interviewee.

No repeat interviews were conducted. A review of the notes showed that after these interviews, data saturation had been reached. All interviews were audio recorded and recordings were referenced to ensure accurate transcription. The first and third authors, who had been trained by the second author in interview methodology, conducted all interviews. Transcripts were not returned to participants; however, results were sent to the interpreters to confirm accuracy. Initial findings were shared with the community at a community gathering to obtain feedback. School enrollment and exam results were also obtained from the school’s records.

### Focus-Group Approach

The first author conducted the FGDs and was trained in facilitation using The Focus Group Kit [37]. The first author had been integrated into the school community for over a month prior to starting FGDs. To ensure open discussion, FGDs were conducted according to gender, grade, and OVC status, yielding a total of six FGDs. The FGDs took place in a private setting at school, intentionally not within a classroom, to foster an open dialogue. At the outset of the FGD, the researcher explained the purpose of the study, and all participants gave verbal assent. The participants were instructed that the discussion could be held in any language and typically English, with intermittent Kiswahili, was used. Participants were informed that all information was confidential and that if they felt uncomfortable at any time they could choose to skip the question or leave the discussion. All FGDs were audio recorded with permission.

Local high school graduates acted as moderators; a female moderator was used for the female FGDs and a male for the male groups. Both moderators were fluent in Kimarachi, Kiswahili, and English and were given moderation training by the first author. The first author acted as a note taker to capture the key issues raised. Each discussion lasted for 45 to 60 minutes. In order to standardize the FGDs, a broad open-ended question guide was formulated. Topics included: benefits of education, reasons for dropout and absenteeism, family and community support of education, and feeding program impacts. For the female groups, menstruation was also included as a topic.

At the conclusion of the FGDs, the moderator and researcher debriefed the FGD to ensure that the notes were comprehensive and accurate. A review of the notes showed that after these six FGDs, data saturation had been reached. Transcripts were not returned to participants.

### Analysis

Key informant interview and student FGD qualitative data were independently analyzed using inductive thematic content analysis [38]. The first and third authors coded the data using semantic themes and sub-themes to ensure consistency and accuracy [38]. Open-ended question responses from guardians were not blocked during analysis, as most guardians were caring for both OVCs and NOVCs, and girls and boys. As such, it was impossible to separate these responses according to OVC status or gender. Given FGDs were conducted according to OVC status and gender, these data were already blocked as necessary.

Household and guardian level quantitative data were blocked by OVC status and gender, and results from these groups were compared to investigate differences in the number of days absent. Chi-square tests were used to compare OVC versus NOVC status and girls versus boys for proportional data including guardian category. Student’s T-tests were used to compare OVC and NOVC status and guardian education attained for normally distributed data including self-reported household income. Poisson regressions were used to compare OVC versus NOVC status, girls versus boys, and guardian category for count data including number of school days absent and number of school days absent for particular reasons. Significance level was set at α<0.05 and analysis was conducted using Statistix version 9 (Tallahassee, Florida, USA).

### Ethics Statement

Ethics approval was obtained from the University of Guelph under REB#14JN026. Guardians provided voice recorded verbal consent for the interviews and for inclusion of their child in the FGDs, and students provided verbal assent. Verbal consent was used to maintain a consistent methodology regardless of guardian literacy. Participants were provided study information including risks, benefits, and contact information for researchers and ethics board contacts.

## Results

### Demographics of Respondents

Interviewed guardians were on average 39.1 (SD = 12.3) years of age, with 75.0% relying solely on subsistence farming as an income-generating activity. Average income was 119.53 (SD = 164.46) KSH (1.33 USD) per day, and was positively associated with guardian education. There was no significant household income differential between OVCs and NOVCs. The average grade reached in school by guardians was 6.6, and 81.3%, 6.25%, and 6.25% of guardians cited school fees, being orphaned, and males being favoured over females, respectively, as their own reason for dropout. There was a wide disparity in education level of guardians: 9 (14.1%) had never attended school, 30 (46.8%) had attended but not completed primary school, 10 (15.6%) had completed primary school but not attended secondary school, and 15 (23.4%) had attended at least some secondary school.

Most OVCs (75.0%) whose father had died still lived with their mother, while only 30.0% of OVCs whose mother had died still lived with their father. Grandparents accounted for 21.9% of the OVCs’ guardians and 12.5% of the NOVCs’ guardians (Table 1). Among the 36 deceased parents, 36.8% of deaths were attributed to unidentified sickness, 21.1% to HIV/AIDS, 15.8% to malaria, and the remaining to road accidents, childbirth, 2007 post-election violence, or unidentified reasons (26.3%). As HIV/AIDS is a taboo topic of discussion in the Luhya culture, it is likely that many of the deaths within the “unidentified illness” category were caused by HIV/AIDS.

**Table 1:** Primary caregiver (guardian category) and parental loss among study participants, Bwaliro village, 2014.

Student Category	Mother	Father	Grandparent	Aunt/Uncle	Sibling	Other wife	Total Students
OVC^a^- Father deceased	12	0	2	2	0	0	16
OVC- Mother deceased	0	3	4	1	1	1	10
OVC- Vulnerable^b^	1	0	0	0	0	0	1
OVC- Both parents deceased	0	0	1	4	0	0	5
NOVC^b^ ^,^ ^c^	27		4	0	0	1	32

^a^ Orphan and Vulnerable Children

^b^ Non-OVC

^c^ These students had both parents

### Perceived value of education

All guardians personally valued education “a lot”; however, only 75.4% of guardians believed that the entire community valued education. All of the female FGDs and one of the male FGDs disclosed that on average, one in five of their peers were not supported in attending school. One key informant stated that, “The growing view of education as a driver of the economy has resulted in about 80% of parents sending their kids to school. Of the remaining parents, half support education but can’t bring their kids to school due to money or health reasons. The others don’t support education because they don’t understand the value” (Key informant 1). All other key informants agreed that most community members support education, and those that do not are “ignorant about its value” (Key informant 3).

While only one guardian mentioned the educational gender disparity, all female FGDs and key informants described the priority often placed on a males’ education. Female students described that girls were often expected to stay at home to complete household chores: “Only when I can’t finish the work at home or in the *shamba* are my brothers told to stay home” (FGD 2, Participant 3, NOVC). Similarly, half of the FGDs and all key informants described that OVCs living with a relative were expected to stay home to provide labour rather than children born to the household head: “If it is harvest season, my [OVC] brothers should help my father in the *shamba*” (FGD 1, Participant 4, NOVC).

Guardians, FGD participants, and key informants alike described community security, job opportunities, and improved personal future, as benefits obtained from attending school. Only three (4.7%) guardians cited a relationship between education and farming ability and health (Table 2). During female FGDs, the predominant benefits discussed were improved family care, health and nutrition. Conversely, male students were more likely to cite job opportunities and improved living standard as benefits. Key informants mentioned that, “educated children are capable of breaking through out-of-date social norms” (Key informant 2) and that education allows children to “cross the bridge from a nobody to a somebody” (Key informant 6).

**Table 2:** Benefits of education perceived by guardians, Bwaliro village, 2014.

Benefit	Total Frequency	Total Percentage (%) (N = 64)
Improved community security	45	70.3
Job opportunities	23	35.9
Improved personal future	21	32.8
Knowledge acquisition	16	25.0
Improved self-reliance	12	18.8%
Improved family care	10	15.6
Improved literacy	5	7.8
Improved communication ability	4	6.3
Improved livestock care and/or permaculture techniques	3	4.7
Improved health and nutrition	3	4.7
Other benefits	14	21.9

### Determinants of dropout and children not enrolled

When asked about primary school enrollment, 35 (54.7%) guardians believed that all community school-aged children were enrolled in school. The other 29 guardians believed that financial reasons and guardians not valuing education were the largest determinants of low enrollment (Table 3). One key informant described that “there is a universal hierarchy of need: food, then shelter, then clothing, then education” (Key informant 1). All students and guardians agreed that these basic needs must be satisfied in order, explaining, “if the family cannot eat or sleep, of course children will not attend school!” (Guardian 54).

**Table 3:** Reasons that children are not enrolled in school as perceived by guardians, Bwaliro village, 2014.

Reason Not Enrolled	Total Frequency	Total Percentage (%) (N = 29)
Financial reasons	19	65.5
Guardians don't value education	7	24.1
Careless guardians	7	24.1
Health reasons	6	20.7
Children unwilling	2	6.9
Peer influence	1	3.8
Alcoholic guardians	1	3.8

No guardians attributed low enrollment to female-related reasons, including menstruation, early marriage, or pregnancy, while all female FGDs and key informants cited these reasons. Girls believed that one in eight of their female classmates dropped out due to menstruation. One participant summarized: “We mostly just fold up cloths to handle the mess, because we are never told what to do and are embarrassed to ask for help” (FGD 2, Participant 1, NOVC). Key informants described that female OVCs living with grandparents were the least likely to be enrolled because of the “tradition of girls staying home to work while boys attend school” (Key informant 4).

### Determinants of Absenteeism

Within the two weeks prior to interviews, 49 of the 64 students had been absent. With 1.59 (SD = 1.31) days on average missed per two weeks, there was an absence rate of 14.2%. Malaria was the leading cause of absence, accounting for over one third of the days missed (Table 4). There was no significant difference in the total number of days missed according to OVC status or guardian category. However, OVCs were 3.8 times (p = 0.01, CI 1.24, 11.80) more likely to be absent due to lack of uniform or money, while NOVCs were 3.0 times (p = 0.04, CI 1.04, 8.65) times more likely to be absent due to malaria.

**Table 4:** Causes of school absences in the two weeks prior to the interview as determined by the guardian, Bwaliro village, 2014.

Reason	OVC^a^ Students (n)	NOVC^b^ Students (n)	Total Students (n)	Total days missed	Total days missed (%) (N = 101)
Malaria	8	16	24	40	39.6
Lack of exam fees, books, or pens	17	7	24	24	23.8
Unspecified illness	10	6	16	18	17.8
Typhoid	1	3	4	9	8.9
Jiggers	1	2	3	4	4.0
Sent away due to misbehaviour	1	0	1	1	1.0
Peer influence	0	1	1	1	1.0
Wake up late	0	1	1	1	1.0
Menstruation	0	1	1	3	3.0

^a^ Orphan and Vulnerable Children

^b^ Non-OVC

Only one guardian mentioned that their child was absent due to menstruation; female FGD groups, however, estimated that on average, 25% of their peers did not attend school when menstruating. Girls then described that the remaining 75% might stay home after lunch due to the discomfort caused by wearing homemade sanitary pad alternatives. The negative impact of menstruation on classroom concentration due to discomfort or the worry of leakage was widely discussed in FGDs: “You never know if you put in enough cloth, so you are always scared that there will be marks on your dress. Because of that, it is so hard to listen to the teacher and finish your papers” (FGD 3, Participant 2, OVC). Girls also unanimously agreed that if the family was hosting visitors, they were forced to stay home to help with housework. Male students attributed absences primarily to exam fees and sickness.

### What Children Are Doing When Not in School

When not in school, all participant groups reported that many children would be helping in the *shamba* with farming, demonstrating the value of their labour. Nineteen (30%) guardians reported that children would be idling around the village, which was perceived to contribute to crime (Table 5). Key informants and FGD participants described that children often leave school in search of food, because: “Some days if I don’t get breakfast or lunch, I can’t concentrate so I just leave to go find some avocados growing on trees” (FGD 5, Participant 2, NOVC).

**Table 5:** Activities conducted by children not in school as reported by guardians, Bwaliro village, 2014.

Activity	Total Frequency	Total Percentage (%) (N = 27)
Farming	13	48.1
Idling	8	29.6
Playing	6	20.7
Helping with housework	6	20.7
Stealing/Causing trouble	4	13.8
Herding cattle	4	13.8
Working for another family	4	13.8
Collecting firewood	2	6.9
Fetching water	2	6.9

### Suggested Mitigation Strategies

When asked to list potential strategies to improve enrollment and attendance, 24 (37.5%) of the guardians could list no strategies. Most guardians, key informants, and FGD participants believed that informing guardians about the benefits of education would be effective. All key informants and 16 (40.0%) guardians proposed school-feeding program expansion as a solution (Table 6). It was described as “putting a worm on the hook of education, immediately fish approach the hook in hunger” (Key informant 3). Guardians also believed that financial support in the form of providing exam fees, pens, or uniforms to the students would encourage better enrollment and attendance. One guardian described that, “If schooling wasn’t so expensive then all children will attend, but with large families and no income, it is difficult to buy all of the supplies for all of the children” (Guardian 3).

**Table 6:** Mitigation strategies for improving school attendance suggested by guardians, Bwaliro village, 2014.

Strategies	Total Frequency	Total Percentage (%) (N = 40)
Community education	20	50.0
Feeding program expansion	16	40.0
Financial support (provision of exam fees, pens, uniforms)	16	40.0
Improved school results	6	15.0
Police enforcement of truancy law	4	10.0
Provision of mosquito nets or malaria treatment	3	7.5
Other	3	7.5
Improved school-community relations	2	5.0
Sponsorship of students to attend high school	2	5.0
Improved efforts from guardians	2	5.0
Improved home nutrition	1	2.5

All female FGDs and key informants discussed that the provision of sanitary pads would substantially improve enrollment and attendance levels, for “if we didn’t have to use old cloths, we would not worry or have discomfort, so we could stay at school” (FGD 3, Participant 1, OVC). One student suggested that, “upper primary girls donate shillings at the beginning of each term so the head teacher can buy us napkins” (FGD 2, Participant 4, NOVC). Other students agreed with this suggestion, provided it was not mandatory, as some students have already established working alternatives.

### Impacts of School Feeding Program

Most guardians (95.5%) who had a child who was fed at school observed substantial changes. The most common reported change was improved health demonstrated by fewer visits to the dispensary and a healthier weight for the child’s height. The feeding program also reportedly had a substantial impact on happiness: “No one fights over food anymore, so everyone is happy and healthy” (Guardian 39). School performance and energy levels also increased (Table 7). All but four guardians stated that their child would still attend school regardless of the feeding program. However, among the guardians who confirmed that their child would still attend, there was discussion that some students would not return after lunch: “without lunch at school, my children would not want to attend because they would want to go climb trees to find fruit” (Guardian 26). When asked what the child was fed on the most recent non-feeding day, 20 students were not fed, and only five (7.8%) students were fed *githeri* or *omena*, the only meals providing significant sources of protein (Table 8).

**Table 7:** Changes in the children since the feeding program began as perceived by guardians, Bwaliro village, 2014.

Observed Change	Total Frequency	Total Percentage (%) (N = 44)
Improved health	35	79.5
Improved performance	30	68.2
Improved happiness	22	50.0
Improved energy level	12	27.3
Improved efforts	5	11.4
Improved concentration/retention	5	11.4
Improved muscle development and strength	4	9.1
Elimination of time spent travelling home for lunch	2	4.5
Reduced sugar cane theft	2	4.5
No change	2	4.5
Improved community at school	1	2.3
Reduced stress	1	2.3

**Table 8:** Mutually exclusive lunch meals given to children on non-feeding days, Bwaliro village, 2014.

Meal	OVC^a^ Frequency n (%) (N = 32)	NOVC^b^ Frequency n (%) (N = 32)	Total Frequency n (%) (N = 64)
Ugali and local vegetables	12 (37.5%)	19 (59.4%)	31 (48.4%)
Nothing	13 (40.6%)	7 (21.9%)	20 (31.3%)
Boiled potatoes	4 (12.5%)	1 (3.1%)	5 (6.3%)
*Githeri* (boiled maize and beans)	1 (3.1%)	0 (0%)	1 (4.7%)
Wheat porridge	1 (3.1%)	1 (3.1%)	2 (3.1%)
Roasted maize	0 (0%)	1 (3.1%)	1 (3.1%)
*Omena* (dried minnows)	0 (0%)	2 (6.3%)	2 (3.1%)
Tea	1 (3.1%)	0 (0%)	1 (1.6%)
Rice	0 (0%)	1 (3.1%)	1 (1.6%)

^a^ Orphan and Vulnerable Children

^b^ Non-OVC

Students in FGDs discussed that most upper primary students stayed at school during lunch, regardless of whether they were fed, to study. Therefore, most NOVC upper primary students attended school for ten hours without eating. The students’ main concerns were their decreasing concentration and energy levels as the day progressed: “during many afternoons I find myself thinking that I should have gone to pick some *loliondo* [olive-like fruit] at lunch” (FGD 3, Student 2, NOVC). OVCs who were fed expressed their increase in energy, attention, and retention. One student also mentioned that, “There is less stress because I know that me and my brothers and sisters will get lunch if we did not have breakfast” (FGD 6, Participant 3, OVC).

One key informant described that since the feeding program began, “concentration, attention, and energy levels have all improved” (Key informant 2). Since the feeding program began, enrollment increased from 685 students to 960 students. Attendance on feeding days was near perfect; however, it remained a concern on non-feeding days. Additionally, since the feeding program was established, the average cumulative grade 8 national examination (Kenyan Certificate of Primary Education) result increased by 5.9% points. Given no other significant changes had been made at the school during these years, key informants believed these changes were a direct result of the feeding program, reporting that, “students now do not want to leave school, as it has become a safe place for fun, learning, and feeding, which satisfies all of their needs” (Key informant 3). The nurses at the local dispensary also stated that malnutrition for school-aged children had dropped from the fourth most treated condition in 2009 to the sixth most treated in 2013.

## Discussion

Our student sample had an absence rate of 14.2%; this rate is slightly higher than Kenya’s average of 11.4%, which could be attributed to higher absence rates in poor, rural areas [15–16]. Contrary to other findings, absent days for OVCs did not differ significantly according to guardian category [19]. However, orphans whose fathers had died tended (p = 0.09) to miss more school than orphans whose mothers had died, which may be attributable to children needing to provide the farming labour typically completed by men. Household income between OVCs and NOVCs did not differ significantly, concurring with Bicego et al.’s findings that from an economic standpoint OVCs are not more disadvantaged than NOVCs [20]. However, OVCs were 3.8 times (p = 0.01, CI 1.24, 11.80) more likely to stay home because of lack of uniform or money, indicating an unequal distribution of funds within these households, where children born to the guardian were given priority for resources. This indicates that OVCs are particularly at risk of being excluded from education for financial reasons. In agreement with Beegle et al. (2004), students mentioned that orphans were expected to stay home before biological children. This may contribute to the enrollment difference of 3.8% between OVCs and NOVCs aged 10 to 14 in Kenya [21,34]. It is therefore crucial that mitigation strategies target the growing OVC population, thereby ensuring that all children have equal access to education [39].

There were continued differences in responses when guardians and FGD participants were asked about the value attributed to education. While only 14.8% of guardians believed that some guardians did not support their children in school, many FGD groups mentioned this lack of support, which may indicate that guardians are unwilling to admit to the lack of support. This discrepancy between guardian and student views was particularly evident in regard to the gender disparity in education. While four guardians mentioned that they themselves had been kept at home due to the prioritization of male education, only one guardian acknowledged that this disparity persists indicating that it is overlooked or believed to be unimportant. Conversely, all female FGD groups stated that girls must stay home to complete chores while their brothers attend school and that the secondary education of boys was greatly prioritized over that of girls. As such, girls’ participation and completion levels remain inferior to those of boys, partly due to household chores, illiterate guardians, gender stereotyping, and factors that influence only girls, including teenage pregnancy and menstruation [12]. In order to achieve universal primary education and gender equality, it is important to address this disparity [7]. The increasing value of education throughout Bwaliro community is a promising development, as it slowly reduces the maternal illiteracy that reinforces girls’ exclusion, however further efforts must be made to close this gap [12]. Multiple researchers have suggested campaigns to transform the minds of guardians from prioritizing boy education as a solution to this ongoing issue [12,40].

Our results showed a positive relationship between highest grade attained by the guardian and income. This finding concurs with Kenya National Bureau of Statistics’ report that poverty drops from 65.5% for household heads with no education to 51.5% for those with primary education and to 27.2% for those with secondary education [33]. Despite this relationship, only 35.9% of guardians recognized that education could reduce poverty by providing better job opportunities. Thus, as found in various developing countries, developing a campaign to teach students and guardians about the benefits and returns of education may serve as an effective strategy to improve enrollment and attendance levels [40–42].

Malaria, accounting for 40% of all absences, has a significant impact on educational achievement. Malaria is the leading cause of morbidity and mortality in Kenya, with almost 70% of the population at risk of infection [2]. Malaria is linked to low educational attainment due to poorer concentration, attendance, and cognition, meaning that programs aiming to prevent malaria in students are crucial to improving enrollment and attendance levels [43]. NOVCs were 3.0 times (p = 0.04, CI 1.04, 8.65) times more likely than OVCs to be absent from school due to malaria suggesting that guardians are more likely to keep their own children home from school if suffering from malaria, as compared to OVCs. Various malaria prevention and treatment strategies have been tested throughout Kenya, and have been found to cost-effectively reduce malaria prevalence suggesting that such options should be explored in Bwaliro [28,43–44]. Educational interventions can also engage pupils in preventative actions at school and home, resulting in decreased prevalence of malaria and other illnesses [45–47]. To this end, there is a reciprocal relationship between malaria prevalence and primary school attendance, in which improved attendance and educational interventions may serve as a realistic malaria control measure [45–47].

The cost of primary school figured prominently as a determinant for dropout and absenteeism. Enforcement of fee policies leads to long absences, occasionally resulting in dropout [48]. Therefore, reducing the primary school costs may improve the issues of dropout and absenteeism. Provision of a uniform, school fees, a daily feeding program, and a school-based helper in western Kenya reduced absenteeism, school dropout, and early marriage rates [10,18]. Such studies found that girls provided with the opportunity to stay in school were also more likely to wait to engage in sexual intercourse, given that sex had higher costs and lower benefits [6,49]. This would therefore also help to reduce HIV transmission and teenage pregnancy [6,17]. Thus, lower education costs would likely lead to increases in school enrollment and attendance while simultaneously improving gender equity and decreasing HIV/AIDS and teenage pregnancy levels.

Menstruation is also a significant barrier preventing girls from completing their primary education. Mirroring our findings, studies in other areas of western Kenya also found that menstruation exacerbates school dropout, poor attendance, and poor concentration in class [22–23]. The World Bank estimates that poor menstrual management results in girls missing 10% to 20% of their school days [16]. Participants of FGDs estimated that 12.5% of girls would drop out and of the others, 25% would be absent due to menstruation. Menstruation was also acknowledged to contribute to low concentration and efforts in class due to the associated shame and fear. Girls in grades six to eight missed on average 2.0 days in a two-week period, while boys in these grades missed only 1.1 days. Conversely, in the younger grades, boys tended to be absent more often (1.95 days) compared to girls (1.3 days) indicating that the differential in older grades may be attributable to menstruation. In rural Kenya, commercial disposable sanitary pads are often either unaffordable or rationed, and due to the limited school and parental support, girls often keep their menses secret from their own mother for fear of being shamed [22]. Therefore, most girls improvise alternatives using old clothes or pieces of bedding [22–23]. These improvised solutions are suboptimal and lack absorbency, contributing to chafing and the fear of leakage that decreases concentration in class [22]. Girls often miss school to bathe, avoid teasing, or due to physical symptoms or lack of items to manage their menstruation [22]. These negative impacts have been recognized and various interventions have been piloted and found to improve attendance, concentration, confidence, and class participation [23,26–27]. In 2009, the government of Kenya pledged to increase funding for water, sanitation and hygiene programs in schools due to the recognized negative impact of poor infrastructure on young girls in particular [50]. However, Alexander et al. (2014) found that these efforts, combined with those of NGOs, remain insufficient in addressing the needs of primary schoolgirls [51]. These policies therefore require further revision to investigate better strategies to allocating funds so as to better serve the needs of these girls. Additionally, in our study, only one guardian mentioned menstruation as a determinant of absenteeism. This suggests that educating guardians about menstruation would be worthwhile to increase parental support and awareness. Previous studies report that reusable pads, deemed acceptable by Kenyan schoolgirls, are 11 times less expensive than a year’s supply of commercial disposable pads [27]. As such, after data collection was complete, a reusable pad program was implemented at Bwaliro Primary School in which girls could purchase reusable pads from the principal after having received appropriate training from a school staff member. All guardians were made aware of the program and were generally supportive of the students purchasing pads. A follow-up study investigating the impact of this program on absenteeism would be worthwhile to evaluate the success of the program and determine if an alternate program using low-cost biodegradable pads may be more effective [27].

Guardians, FGD participants, and key informants agreed that the established feeding program has significantly improved enrollment, attendance, concentration, school performance, and happiness. Given that the program feeds all children in grade 3 and under, and the fact that most households consist of a mix of OVCs and NOVCs, it was impossible to compare the impact of the program between the two groups. The reported positive impacts concur with many other feeding programs implemented in other developing countries [52–55]. A Nepalese study found that students suffering from economic or nutritional distress are more likely to be absent from school, which concurs with the general agreement that a child requires food, clothing, and shelter, before education becomes a priority [56]. This finding strengthens the case for school feeding as an encouragement to attend school. The positive effect of feeding programs on attendance can be attributed to providing both guardians and students with an incentive for children to attend school, and to better health that reduces incidence of sickness [56]. While the feeding program has greatly improved school attendance, home nutrition must also be addressed since nutritional status is an important determinant of school participation [31]. Better-nourished children tend to have better school attendance records than those who are malnourished [31]. Thus, nutrition seminars aiming to improve community nutrition by using locally available food may also help to improve attendance. The Kenyan government also recognizes that inadequate nutrition is a challenge to community and economic development [4]. It therefore hopes to begin providing lunch to disadvantaged children, reintroducing school milk programs, and implementing Home Grown School Meals Programmes to ensure feeding program sustainability [4]. A timeline for this project, however, was not provided [4]. Expansion of the school feeding program to include all students and feed everyday should be evaluated, as the feeding program has been observed to positively impact both OVCs and NOVCs, with attendance reportedly higher on feeding days.

One challenge encountered throughout the study was the subjectivity of face-to-face questionnaires; while participants were encouraged to answer honestly, they sometimes sought the “correct” answer. To minimize this impact, researchers ensured prior community integration and used local interpreters. To further decrease the potential of responder bias, no Children of Bukati affiliate or benefactor was involved in data collection. Prior to beginning interviews and FGDs, it was made clear that the researchers had no influence on program funding. While responder bias is always a concern, we believe that we took the necessary measures to reduce the potential impacts. In addition to responder bias, the taboo nature of certain topics, including menstruation and HIV/AIDS, may have influenced responses. It is also important to note that our sample consisted only of students currently enrolled at Bwaliro Primary School and did not include unenrolled children. In light of these limitations, this data can only be generalized to rural villages in western Kenya and is most applicable in villages where primary schools already have established feeding programs, as these programs significantly decrease the impact of food insecurity on school enrollment and absenteeism.

## Conclusion

The competencies and skills students obtain through quality education have the potential to improve many dimensions of Kenyan life and achieve multiple MDGs. Kenya’s government has aimed to improve primary school attendance by implementing free primary education; however, many barriers continue to prevent children from attending school. Our findings indicate that malaria, menstruation, and lack of finances are among the most significant determinants of primary school dropout and absenteeism. These factors disproportionately affected OVCs and female students, indicating that mitigation strategies should specifically target these populations. The most promising mitigation strategies suggested included campaigns to educate students and guardians about the benefits of education, provision of malaria treatment or prevention, reducing education costs, providing sanitary pads, and expanding the established feeding program. Further research is needed to assess the cost effectiveness of these programs in this context. Establishing such programs would improve enrollment and attendance, thus increasing the number of children and youth who are learning the important competencies and skills offered at school.
